# Molecular Detection of Virulence Factors in *Salmonella serovars* Isolated from Poultry and Human Samples

**DOI:** 10.1155/2023/1875253

**Published:** 2023-03-02

**Authors:** Kelly Johanna Lozano-Villegas, María Paula Herrera-Sánchez, Mónica Alexandra Beltrán-Martínez, Stefany Cárdenas-Moscoso, Iang Schroniltgen Rondón-Barragán

**Affiliations:** ^1^Poultry Research Group, Laboratory of Immunology and Molecular Biology, Faculty of Veterinary Medicine and Zootechnics, University of Tolima, Santa Helena Highs, Postal Code 730006299, Ibagué, Tolima, Colombia; ^2^Immunobiology and Pathogenesis Research Group, Laboratory of Immunology and Molecular Biology, Faculty of Veterinary Medicine and Zootechnics, University of Tolima, Santa Helena Highs, A. A 546, Ibagué 730006299, Colombia

## Abstract

Salmonellosis is a common infectious disease in humans caused by *Salmonella* spp., which in recent years has shown an increase in its incidence, with products of avian origin being a common source of transmission. To present a successful infective cycle, there are molecular mechanisms such as virulence factors that provide characteristics that facilitate survival, colonization, and damage to the host. According to this, the study aims to characterize the virulence factors of *Salmonella* spp. strains isolated from broilers (*n* = 39) and humans (*n* = 10). The presence of 24 virulence genes was evaluated using end-point PCR. All the strains of *Salmonella* spp. isolated from broiler chickens revealed presence of 7/24 (29, 16%) virulence genes (*lpfA*, *csgA*, *sitC*, *sipB*, *sopB*, *sopE*, and *sivH*). Regarding the strains isolated from cases of gastroenteritis in humans, all strains contained (14/24, 58, 33%) virulence genes (*lpfA*, *csgA*, *pagC*, *msgA*, *spiA*, *sitC*, *iroN*, *sipB*, *orgA*, *hilA*, *sopB*, *sifA*, *avrA*, and *sivH*). In summary, the presence of virulence genes in different strains of *Salmonella* isolated from broilers and humans could be described as bacteria with potential pathogenicity due to the type and number of virulence genes detected. These findings are beneficial for the pathogenic monitoring of *Salmonella* in Colombia.

## 1. Introduction

Salmonellosis is a foodborne disease with the greatest impact worldwide on both humans and animals [1, 2]. This disease is caused by the *Salmonella* in which more than 2,700 serotypes have been reported so far [3]. In humans, the consumption of chicken meat and eggs that were contaminated is conduced to develop the disease because they are considered the main reservoir and vehicle of Salmonella [1, 4]. Moreover, food contamination could occur in various stages of the food chain such as production, distribution, and sale [5]. The serotype, infective dose, virulence factors, and host immunity will influence the disease's clinical presentation [6]. Salmonellosis in humans is characterized by symptoms such as acute fever, abdominal pain, diarrhea, nausea, and vomiting; however, immunocompromised people and children under 5 years of age and older adults can present severe symptoms [7, 8]. In 2018, the EU member states reported 5146 foodborne outbreaks where 33% correspond to illnesses caused by *Salmonella* [7].

Host-pathogen interactions in bacteria can modulate the expression of some genes to adapt to the environment, influencing their ability to cause illness [9]. Therefore, the virulence genes facilitate the survival, colonization, and damage of the host [10]. Expression of virulence genes will initiate when *Salmonella* spp. faces the hostile environment of the hosts' gastrointestinal tract compound of a wide variety of conditions such as osmolarity, oxygen tension, and pH which favor interaction with the target cell during pathogenesis [2]. The molecular mechanisms of pathogenicity used by *Salmonella* involve genes, grouped in regions called pathogenicity islands that provide new characteristics that allow it to undergo a successful infective cycle [11]. These genetic segments linked to virulence functions are known as Salmonella pathogenicity-island (SPI) and Salmonella has 24 identified [12]. In addition, SPI could be transmitted between bacteria by horizontal gene transfer and is related to virulence mechanisms such as host colonization, capsules, toxins, invasiveness, biofilm, fimbriae, flagella, serotype conversion, and secretion systems [12–14].

Overall, bacterial virulence factors are critical elements for systemic infections [11]. As a result, the pathogenicity of *Salmonella* has been associated with the number and type of virulence genes present in the chromosomal SPIs [15]. For example, genes such as *SopB*/*SigD* and *SopE2* allow a rapid internalization of the bacteria playing an important role in *Salmonella* virulence [16]. Moreover, genes involved in the intracellular survival of *Salmonella* play a significant role in systemic disease in humans [17]. Meanwhile, adherence factors like fimbrial operons mediate the attachment of *Salmonella* serovars to epithelial cell lines [18]. Besides, *Salmonella* virulence plasmid plays a crucial role in enhancing the ability of particular serovars to multiply in tissues outside the intestinal tract [19]. Other genes, such as *cdtB*, code for the CdtB subunit considered as a toxin with a possibly important role in the unusually lengthy, persistent, and development of systemic diseases [20, 21].

Despite being a public health concern, there are insufficient studies on virulence factors in *Salmonella* spp. isolates from broilers and humans in Colombia; also, without specific information, it is difficult to predict the success of *Salmonella* control schemes. Thus, it is important to know the genomic particularities in each of the serotypes belonging to this genus; this allows to clarify the bacterial dynamics in the different animal hosts and prevent outbreaks in humans and animals [11]. Thus, the aim of this study was to evaluate the potential virulence of *Salmonella* isolates from poultry and human by detecting the presence of 24 genes involved in virulence and pathogenicity using the polymerase chain reaction (PCR). Accordingly, the results of this study could lay the foundation for further research on public health security and food safety problems caused by *Salmonella* infections in Colombia.

## 2. Materials and Methods

### 2.1. *Salmonella* Strains

In this study, 49 *Salmonella enterica* strains from the Bacterial Strain Collection of the Laboratory of Immunology and Molecular Biology were included, and *Salmonella enteritidis* (ATCC® 13076™) were used as a positive control. The strains were previously serotyped using the Kauffmann−White scheme and correspond to the serotypes, namely, *S. enteritidis* (*n* = 4), *S. typhimurium* (*n* = 2), *S. braenderup* (*n* = 1), *S. newport* (*n* = 1), *S. grupensis* (*n* = 1), and *S. uganda* (*n* = 1) isolated from cases of gastroenteritis in humans [22] and *S. paratyphi* B (*n* = 24) and *S. heidelberg* (*n* = 15) isolated from poultry farms located in the region of Tolima [23] and Santander [24].

### 2.2. DNA Extraction

Fresh bacterial colonies were used for Genomic DNA (gDNA) extraction using the Invisorb Spin Universal Kit (Stratec Molecular, Berlin, Germany) following the protocol suggested by the fabricant and were stored at −20°C until further use. Molecular confirmation of *Salmonella* isolates was done by amplification of a fragment of *invA* gene (accession number M90846.1) by endpoint PCR.

### 2.3. Virulence Genes

The molecular characterization of 24 genes involved in virulence and pathogenicity was conducted using the gDNA of *Salmonella* spp. (Table 1). A single PCR assay was used to detect each one of the 24 virulence genes. Primers and annealing temperature used for PCR are listed in Table 1. The reactions were carried out following the manufacturer's recommendation for the GoTaq® Flexi DNA Taq polymerase (Promega, Madison, WI, United States), 1 *μ*L of DNA, and 1 *μ*L of each primer (10 pmol/*μ*L). The ProFlex™ 3 × 32-well PCR System (Applied Biosystems, Carlsbad, CA, United States) was used to perform the amplification using an initial denaturation for 3 minutes at 95°C, 35 cycles of denaturation for 30 seconds at 95°C, 30 seconds of annealing (Table 1), extension at 72°C, and final extension for 5 minutes at 72°C. The PCR products were detected by electrophoresis in agarose gel using HydraGreen (ACTGene, Piscataway, NJ, United States) as an intercalant agent, and the visualization of the gel was conducted in the gel documentation equipment ENDURO GDS (Labnet International, Edison, NJ, United States).

## 3. Results

### 3.1. Confirmation of *Salmonella*

**Figure 1:**
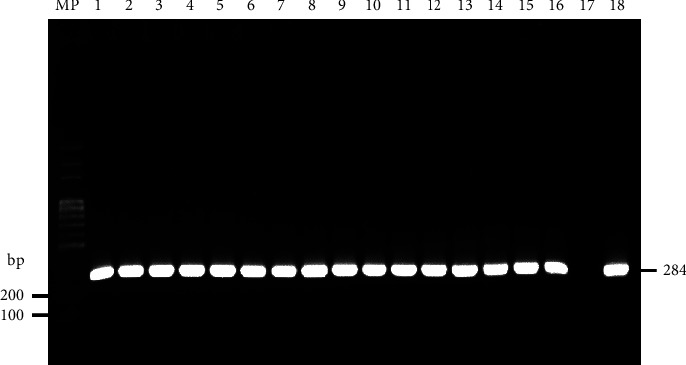
Gel image of the PCR amplification of a DNA fragment from the *invA* gene (284 bp) of representative *Salmonella* strains isolated from broiler farms. MP-100 bp DNA ladder (Solis BioDyne, Estonia); 1–4: *S*. *heidelberg*; 5–9: *S*. *paratyphi* B; 10: *S*. *newport*; 11-12: *S*. *enteritidis*; 13: *S*. *braenderup*; 14: *S*. *uganda*; 15: *S*. *grupensis*; 16: *S*. *typhimurium*; 17: negative control *E. coli* ATCC 25922; 18: positive control *S*. *enteritidis* ATCC 13076.

All *Salmonella* strains amplified the expected DNA fragment of the *invA* gene that was used to confirm the *Salmonella* genus (Figure 1).

### 3.2. Distribution of *Salmonella* Virulence Genes

All the isolates carried the *lpfA*, *csgA*, *invA*, *sivH*, *sopB*, and *sitC* genes (*n* = 49/49). Regarding poultry isolates, the detection rate of the *sopE* gene was 100% (*n* = 39/39), while fimbria-associated genes such as *sefA*, *lpfC*, and *lpfA* were present in 51.3% (*n* = 20/39), 87.2% (*n* = 34/39), and 97.2% (*n* = 34/39). Genes associated with type III secretion systems (TTSS) virulence that function as *orgA*, *prgH*, and *spaN* were found in 71.8% (*n* = 37/39), 74.4% (*n* = 36/39), and 79.5% (*n* = 31/39), respectively, of the poultry isolates that were analyzed (Table 2). The detection rate of an effector protein gene like *avrA* was 97.4% (*n* = 38/39). Gen with regulatory protein function as *hilA* was found in 94.9% (*n* = 37/39). Genes related to survival inside cells functions such as *pagC*, *spiA*, *msgA*, and *tolC* were present in 97.4% (*n* = 38/39), 97.4% (*n* = 38/39), 97.4% (*n* = 38/39), and 92.3% (*n* = 36/39) of the poultry strains that were analyzed. The detection rate of pSLT-mediated virulence genes such as *spvB* was 71.8% (*n* = 37/39). The *iroN* gen was present in 97.4% (*n* = 38/39) of the poultry isolates that were analyzed.

On the other hand, all the isolates from cases of gastroenteritis in humans carried *spiA*, *pagC*, *hilA*, *avrA*, *msgA*, *orgA*, and *iroN* genes (Table 2). The detection rates of *sefA*, *lpfC*, and *lpfA* were 90% (*n* = 9/10), 80% (*n* = 8/10), and 60% (*n* = 6/10), respectively, whilst those for *prgH* and *spaN* genes were 90% (*n* = 9/10) and 80% (*n* = 8/10), respectively. The *sopE* gene was present in 70% (*n* = 7/10) of the human isolates, respectively. The detection rates of *tolC* and *spvB* genes were 60% (*n* = 6/10) and 60% (*n* = 6/10), respectively.

### 3.3. Virulence Gene Patterns in *Salmonella* Isolates

Detection of the twenty-four virulence genes by PCR classified 49 selected *Salmonella* isolates into 21 patterns (Table 2). The virulence gene patterns of cases of gastroenteritis in humans were Enteritidis (III), Braenderup (XXII), Newport (XX), Grupensis (XXV), Uganda (XXIII), and Typhimurium (XXIV).

The pattern III was the most predominant that was detected in 10 isolates (Heidelberg 3, Paratyphi B 4, Enteritidis 2). In poultry farm isolates, pattern II with 21 virulence genes was detected in 8 isolates (Heidelberg 4, and Paratyphi B 4). The pattern I was detected in 6 isolates (Heidelberg 3, Paratyphi B 3). Patterns X, XI, XII, XIII, XIV, XV, XVII, XVIII, and XIX were detected only in serotypes of Paratyphi B. Also, patterns II, IV, VII, and IX were only observed in Heidelberg isolates. Patterns I, III, V, and VIII were observed in poultry isolates.

## 4. Discussion

*Salmonella* species are ubiquitous pathogens that are considered the major agents of foodborne disease worldwide [27, 28]. In *Salmonella* spp., physiological and environmental stimuli drive the expression of virulence genes, which are responsible for the main pathogenic mechanisms in this microorganism [10, 29]. Virulence factors can maximize the fitness of pathogens via host exploitation [30]. Virulence factors are encoded by a number of genes and may be located on *Salmonella* pathogenicity islands (SPI), virulence plasmids (pSLT), bacteriophages, or at another location on the chromosome [25, 27, 31].

A few virulence factors are related with the cellular structure of the bacteria, such as fimbriae [32]. Fimbrial virulence genes represent a major player in pathogenesis by allowing bacteria to interact with host cells [33, 34]. In the present study, all the isolates carried the *lpfA* and *csgA* genes. Similarly, previous studies have found high detection of the *csgA* gene among *Salmonella* serotypes [25, 35]. The *csgA* gene is related to biofilm production and the maintenance of the bacteria in the environment, including inert surfaces [36]. Likewise, the presence of the *csgA* gene is relevant to public health because the *csg* genes in *Salmonella* are related to the ability to produce biofilms, leading to increased drug resistance [37]. In this way, the presence of the *csgA* gene in all the strains could suggest that the *Salmonella* strains could be kept on inert surfaces such as those used in food production, which is relevant to public health. In poultry isolates, the detection rate of fimbria-associated genes such as *sefA* and *pefA* were 51.3% and 74.4%, respectively, lower than the other virulence genes evaluated. Additionally, the detection rate of pefA was 60% in isolates from cases of gastroenteritis in humans. The presence of the *sefA* gene in *Salmonella* isolates is relevant because this gene is a promotor of the *sef* operon, and this operon is a mechanism by which *Salmonella* serotypes can adapt to an increasing number of hosts [25, 38]. Previous studies of virulence gene detection in *Salmonella* Heidelberg isolated from chicken carcasses did not report isolates with the *sefA* gene [25]. For this reason, it is possible that the presence of the *sefA* gene in *Salmonella* Heidelberg isolates could indicate a major virulence of the strains. Furthermore, *sefA* gene has been associated with the serotypes Enteritidis, Moscow, but the horizontal transfer methods allowed other serotypes to obtain different genes than that in the case of this study serotypes such as Paratyphi B and Braenderup, and Typhimurium carried the gene [39]. The absence of the *pefA* gene in some *Salmonella* serotypes is related to the location of the gene, which is plasmidial [40].

The type III secretion system (TTSS) encoded by Salmonella mediates, in a contact-dependent manner, the translocation of effector proteins from the bacterial cytoplasm into the host cell [41]. Some genes of TTSS are related to structure, effector protein, or regulatory protein of these systems [42]. The *sipB*, *invA*, *orgA*, *prgH*, and *spaN* genes are associated to the structure of TTSS, which allows *Salmonella* to invade phagocytic and nonphagocytic cells [40, 43]. The *sipB* and *invA* genes were found in 100% of the isolates that were assessed (*n* = 49/49). The *sipB* gene may play a vital role in *Salmonella* pathogenesis [44]. In the case where detection rates of the *invA* gene were expected, this gene is recognized as a rapid detection agent for the genus *Salmonella*, and this gene also indicates that all the strains are able to produce gastroenteritis and invade the cells [45, 46]. A high prevalence of *orgA*, *prgH*, and *spaN* genes in poultry and human isolates were observed in this study (71–100%). In the same way, previous research has detected *sipB*, *orgA*, *prgH*, and *spaN* genes in *Salmonella* isolates from poultry-related sources [47].

Furthermore, TTSS is employed by *Salmonella* to inject different “effector proteins” into host cells [48]. Each effector protein activates or blocks a specific host cell signaling pathway to establish symbioses or infectious diseases [49]. Some genes that encode the effector proteins are *avrA*, *sopE*, *sopB*, and *sivH* [50]. All the isolates carried the *sivH*, *sopB*, and *sopE* genes whilst the human isolates analyzed were 70%. The *SopB* gene can regulate changes in phosphatidylinositol signaling that could generate chloride secretion by epithelial cells [51]. Thus, the significance of the presence of the *sopB* gene is because of the fact that strains with this gene can cause diarrhea, and this disease leads to the elimination of large numbers of bacteria in the host's environment [52]. Consequently, the possession of this gene could increase the spread of *Salmonella*. High frequency of *sivH* and *sopE* may be explained by the fact that these genes are associated with an island which is unique to *Salmonella* infecting warm-blooded vertebrates [53, 54]. The detection rate of an effector protein gene like *avrA* gene was 97.4% in poultry and 100% in human isolates. AvrA protein plays a critical role in inhibiting inflammation, regulating epithelial apoptosis, and enhancing proliferation during bacterial infections [55–58]. On the other hand, gene with regulatory protein functioning as *hilA* was found in 94.9% in poultry isolates. All the isolates from cases of gastroenteritis in humans carried the *hilA gene.*

Some virulence genes may contribute to survival within the macrophage or intracellular survival, for example, *pagC*, *spiA*, *msgA*, and *tolC* genes [59]. The *pagC* gene is ubiquitously distributed among *Salmonella* serotypes [60]. As a result, prevalence found in *pagC* gene was 97.4% in poultry and 100% in human isolates. The detection rate of the *spiA*, *msgA*, and *tolC* genes was higher than 92.3% in all the poultry isolates that were analyzed. On the other hand, all the isolates from cases of gastroenteritis in humans carried *pagC*, *spiA*, and *msgA* genes. The high frequency of the *spiA* gene in poultry and human samples is considered critical due to the function of the gene that is related to the ability of the *Salmonella* serotypes to produce biofilms [25]. Biofilm is an important public health problem; it enhances resistance to physical forces, the host immune system, and antimicrobials [61, 62]. In this way, *Salmonella* strains with the *spiA* gene would survive longer in poultry farms and could contaminate meat and eggs, where contaminated food is a vehicle in the transmission of *Salmonella* to humans. The detection rate of *tolC* in human isolates was 60%. The *tolC* gene plays a crucial role in the excretion of a wide range of molecules, including antibiotics [63].

The detection rate of pSLT-mediated virulence genes such as *spvB* was 71.8% in poultry and 60% in human isolates, and the frequencies may be explained by the *spvB* gene that is located on virulence plasmids [64]. However, the *spvB* gene that is present in these isolates is relevant because *spv* genes are highly associated with strains that cause nontyphoid bacteremia and disseminated infection in humans [17, 65]. In addition, genes related with iron metabolism such as *iroN* gene that are related to iron acquisition were 97.4% in poultry and 100% in human isolates that were analyzed [66]. Also, the *sitC* gene is another gene that is related to iron metabolism, and this gene encodes an important transporter of iron [67], and all the isolates carried the gene. Previously, the presence of the *spaN* gene was reported, but the *iroN* gene was not associated to bacteria isolated from poultry sources [68]. In the case of *S.* Heidelberg, the fifteen strains carried the two genes. The significance of *iroN* gene cluster that is present on the *Salmonella* isolates is because of the fact that the iron gene that is present represents an adaptation to life at inflamed mucosal surfaces [69]. On the other hand, Webber et al. reported that 88.9% (*iroN;* 112/126) and 79.4% (*sitC;* 100/126) of the *Salmonella* Heidelberg carried the gene [25]. Nevertheless, the presence of the virulence genes does not indicate that the bacteria is pathogenic, it necessarily combined the expression of multiple genes [70]. Finally, we suggest performing other methodologies to confirm the expression of genes or proteins related to virulence factors for a better characterization of each *Salmonella* strains.

## 5. Conclusions

An analysis of the virulence genes of *Salmonella enterica* was conducted to assess its pathogenic potential. In summary, this study provided a better insight into the epidemiology and pathogenicity of *Salmonella* serovars circulating in two Colombia regions. Also, the presence of virulence genes in different strains of *Salmonella* isolated from broilers and humans could describe it as bacteria with potential pathogenicity due to the type and number of virulence genes detected. In this way, we recommend active surveillance to have updated information on the pathogenicity of *Salmonella enterica* strains circulating and preventing outbreaks of *Salmonella* infection.

## Figures and Tables

**Table 1:** Primer sequences for virulence genes in *Salmonella* spp.

Virulence factor	Gene	Primer sequences	Annealing temperature (°C)	Amplicon size (bp)	References
Fimbriae	*lpfC*	F-GCCCCGCCTGAAGCCTGTGTTGC	58	641	[25]
		R-AGGTCGCCGCTGTTTGAGGTTGGATA			
	*pefA*	F-GCGCCGCTCAGCCGAACCAG	59	157	
		R-GCAGCAGAAGCCCAGGAAACAGTG			
	*lpfA*	F-CTTTCGCTGCTGAATCTGGT	46	250	
		R-CAGTGTTAACAGAAACCAGT			
	*csgA*	F-TCCACAATGGGGCGGCGGCG	54	350	
		R-CCTGACGCACCATTACGCTG			
	*sefA*	F-GATACTGCTGAACGTAGAAGG	54	488	
		R-GCGTAAATCAGCATCTGCAGTAGC			
Plasmid	*spvB*	F-CTATCAGCCCCGCACGGAGAGCAGTTTTTA	58	717	
		R-GGAGGAGGCGGTGGCGGTGGCATCATA			
Survival inside cells	*tolC*	F-TACCCAGGCGCAAAAAGAGGCTATC	55	161	
		R-CCGCGTTATCCAGGTTGTTGC			
	*pagC*	F-CGCCTTTTCCGTGGGGTATGC	55	454	
		R-GAAGCCGTTTATTTTTGTAGAGGAGATGTT			
	*msgA*	F-GCCAGGCGCACGCGAAATCATCC	57	189	
		R-GCGACCAGCCACATATCAGCCTCTTCAAAC			
	*spiA*	F-CCAGGGGTCGTTAGTGTATTGCGTGAGATG	56	550	
		R-CGCGTAACAAAGAACCCGTAGTGATGGATT			
Toxins	*cdtB*	F-ACAACTGTCGCATCTCGCCCCGTCATT	57	268	
		R-CAATTTGCGTGGGTTCTGTAGGTGCGAGT			
Iron metabolism	*sitC*	F-CAGTATATGCTCAACGCGATGTGGGTCTCC	58	768	
		R-CGGGGCGAAAATAAAGGCTGTGATGAAC			
	*iroN*	F-ACTGGCACGGCTCGCTGTCGCTCTAT	58	1205	
		R-CGCTTTACCGCCGTTCTGCCACTGC			
Structure, the invasion-associated type III secretion system	*prgH*	F-GCCCGAGCAGCCTGAGAAGTTAGAAA	57	756	
		R-TGAAATGAGCGCCCCTTGAGCCAGTC			
	*spaN*	F-AAAAGCCGTGGAATCCGTTAGTGAAGT	55	504	
		R-CAGCGCTGGGGATTACCGTTTTG			
	*sipB*	F-GGACGCCGCCCGGGAAAAACTCTC	58	875	
		R-ACACTCCCGTCGCCGCCTTCACAA			
	*invA*	F-GTGAAATTATCGCCACGTTCGGGCAA	55	284	
		R-TCATCGCACCGTCAAAGGAACC			
	*orgA*	F-TTTTTGGCAATGCATCAGGGAACA	55	255	
		R-GGCGAAAGCGGGGACGGTATT			
Regulatory protein, the invasion-associated type III secretion system	*hilA*	F-CTGCCGCAGTGTTAAGGATA	50	497	
		R-CTGTCGCCTTAATCGCATGT			
Effector protein, the invasion-associated type III secretion system	*sopB*	F-CGGACCGGCCAGCAACAAAACAAGAAGAAG	57	220	
		R-TAGTGATGCCCGTTATGCGTGAGTGTATT			
	*sifA*	F-TTTGCCGAACGCGCCCCCACACG	58	449	
		R-GTTGCCTTTTCTTGCGCTTTCCACCCATCT			
	*avrA*	F-AGCCTGGCGCTCGCCAAAAA	57	123	[26]
		R-GCGGTCTGCTTTATCGGACGGG			
	*sopE*	F-GAGGGCCGGGCAGTGTTGAC	55	121	
		R-CTTCACGGGTCTGGCTGGCG			
	*sivH*	F-AGCGCGCTGAATGCGGTGAT	55	121	
		R-TCTTGTCGCGCCACAGCAGG			

**Table 2:** Patterns of virulence genes of *Salmonella* isolates obtained from poultry farms and cases of gastroenteritis in humans.

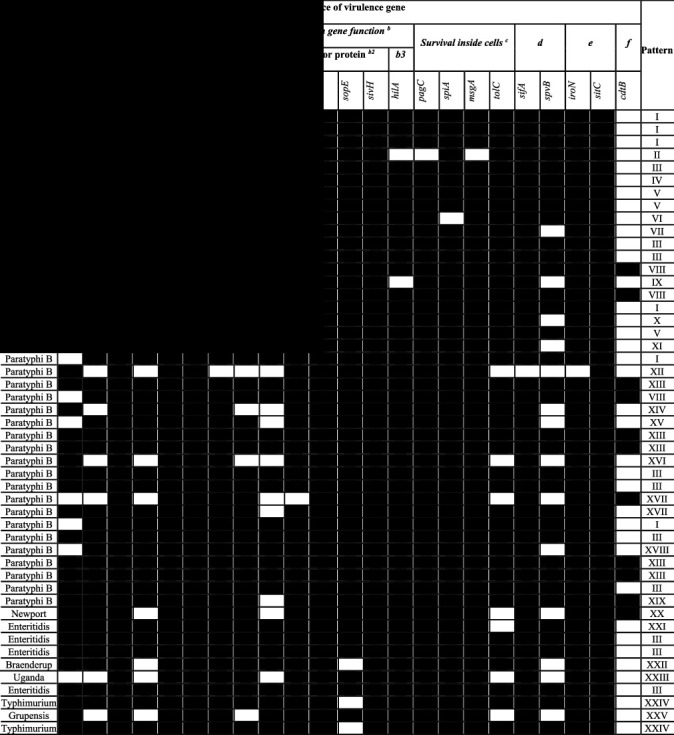

^*∗*^For PCR-based patterns, black area represents a positive result and white area represents negative result for the presence of a virulence gene. Virulence-related function is a Fimbriae gene function; b is type three secretion system gene function, b1 structure, b2 is effector protein, b3 is regulatory protein; c is survival inside cells gene function; d is plasmid gene function, e is iron metabolism gene function, and f is toxins gene function.

## Data Availability

The data were obtained from the study. Also, all the datasets generated or analyzed during this study are included in this manuscript.
